# *The Hospital's* Commission on Light Wines

**Published:** 1907-06-22

**Authors:** Andrè L. Simon

**Affiliations:** Author of *The History of the Wine Trade in England*


### Editor's Letter-Box.

[Our Correspondents are reminded that prolixity is a great bar to publication, and that brevity of style and conciseness of statement greatly facilitate early insertion.]

### *THE HOSPITAL'S* COMMISSION ON LIGHT WINES.

Sir,—The report on light wines published in your last number has attracted considerable attention and, on the whole, very laudatory comment from the trade. The men who drafted it have evidently gone into the matter thoroughly and with a real desire of getting at the truth; this fact has induced me to point out to you a technical error which, I feel sure, members of your Commission will readily recognise when brought to their notice.

They state (p. 290) that champagne gets an addition of liqueur—or dosing; this process, however, is restricted to the sweet champagne, and there is a good deal of champagne sold in this country to which no such liqueur has ever been added.

They state (also p. 290) that all champagne contains a considerable quantity of sugar and that, in the extra-dry champagne they examined, the sugar was very high. As a matter of fact, there are some brands of champagne which are labelled "Extra Dry" in lieu of "Extra Acid," and which contain added sugar, and it is unfortunate that the choice of the Commissioners should have fallen on these for their experiments. At the same time, having had considerable experience in the manufacture of champagne in Reims, and having by me the results of various thoroughly reliable analyses in this country, I have not the slightest hesitation in asserting that there is a great deal of champagne in this country in which practically no sugar will be found.

It would give me great pleasure to send you samples of different brands of champagne for your examination, both of the expensive and cheaper kinds, in which I am absolutely certain that the most conscientious analysis will reveal no added sugar whatever.

I shall esteem it a favour if you will bring the above to the notice of the medical men who drafted the report published by you, assuring them of my willingness to furnish them with any technical information they may desire on the subject,

I am, dear sir,
Yours faithfully,
André L. Simon,
Author of *The History of the Wine Trade in England.*

[We were careful to purchase the champagnes analysed on the open market at an average price for good wines. We publish this letter at once, and the Commission's answer will appear next week.—Ed., The Hospital.]

